# K-Ras Mutations in Non-Small-Cell Lung Cancer: Prognostic and Predictive Value

**DOI:** 10.5402/2012/837306

**Published:** 2012-05-14

**Authors:** Manolo D'Arcangelo, Federico Cappuzzo

**Affiliations:** Istituto Toscano Tumori, Ospedale Civile di Livorno, 57100 Livorno, Italy

## Abstract

Non-small-cell lung cancer (NSCLC) is a heterogeneous disease due to the presence of different clinically relevant molecular subtypes. Until today, several biological events have been identified in lung adenocarcinoma, including epidermal growth factor receptor (*EGFR*) mutations and anaplastic lymphoma kinase (*ALK*) translocations, offering new hopes to patients with metastatic disease. Unfortunately, in approximately 50% of adenocarcinoma and for those harbouring *K-RAS* mutations, the most frequent mutation in Caucasian lung adenocarcinoma, so far no specific drug demonstrated efficacy. The rat sarcoma (*RAS*) genes, including *H-RAS, K-RAS,* and *N-RAS*, encode a family of proteins regulating cell growth, differentiation, and apoptosis. *K-RAS* mutations are present in 20–30% of NSCLC and occur most commonly, but not exclusively, in adenocarcinoma histology and life-long smokers. Although in colorectal cancer patients *K-RAS* mutations represent a validated negative predictive biomarker for treatment with anti-EGFR monoclonal antibodies, their role in selecting specific treatment for NSCLC patients remains undefined. Aim of the present paper is to critically analyze the prognostic and predictive value of *K-RAS* mutations in NSCLC.

## 1. Introduction

In 2011 non-small-cell lung cancer (NSCLC) remains the principal cause of cancer-related death worldwide, accounting for more than one million deaths per year [1]. Therapeutic progresses have signed out the last decade, but median survival for patients in advanced stage is still disappointing [2]. NSCLC accounts for 80% of lung tumors, including adenocarcinoma in 35–40% of cases, squamous cell carcinoma in 25–30%, and large cell carcinoma in 10–15%. For many years we treated metastatic NSCLC with the same regimens, irrespective of any clinical or biological characteristics. Today, histology seems a relevant parameter for defining the best regimen, with new agents, such as pemetrexed and bevacizumab, effective and safe only in non-squamous populations [3, 4]. During the last few years, improvement in the knowledge of lung cancer biology led to identification of molecular events crucial for tumor cell survival. Cancer cell survival might depend on the expression of a single-mutant oncogene according to a model called “oncogene addiction” [5, 6]. In NSCLC a number of driving mutations have been identified, including Epidermal growth factor receptor (*EGFR)* mutations*, KRAS *mutations, *HER2* mutations and *EML4-ALK* translocations. Since their identification in 2004, activating *EGFR* gene mutations have emerged as the most relevant predictor of response to a class of compounds, the *EGFR* tyrosine kinase inhibitors (EGFR-TKIs) gefitinib and erlotinib. Six phase III randomized trials demonstrated that patients harboring activating *EGFR* mutations benefit more from EGFR-TKIs than from standard platinum-based chemotherapy at least in terms of response rate (RR), progression-free survival (PFS), toxicity profile and quality of life [7–12]. Randomized phase III trials in the maintenance setting (SATURN and ATLAS), in second-line versus chemotherapy (INTEREST and TITAN) and versus placebo (BR21) confirmed the high efficacy of EGFR-TKIs in the presence of activating *EGFR* mutations [13–17]. Today in patients harbouring an *EGFR* mutation, gefitinib or erlotinib represent the best therapeutic option irrespectively of treatment line. Nevertheless, large randomized clinical trials demonstrated that erlotinib could produce a modest benefit even in the *EGFR* wild-type population [13, 14].

Therefore, a relevant issue in clinical practice is the identification of *EGFR* wild-type patients that could benefit or that could be excluded from an EGFR-TKI therapy. Unfortunately, at present, there is no single biomarker that could be used for precluding the treatment to any patient, including *K-RAS* mutations [14]. Although in colorectal cancer *K-RAS* mutations are the most useful biomarker for selecting patients who are candidate for treatment with anti-EGFR monoclonal antibodies, cetuximab or panitumumab, its role in NSCLC as prognostic or predictive marker is less defined [18]. The aim of the present paper is to analyze the role of *K-RAS* mutations in NSCLC.

## 2. *RAS* Mutations in NSCLC

**Figure 1:**
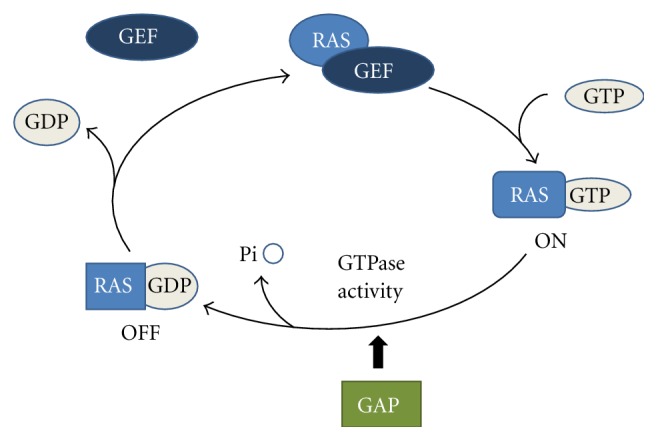
Ras activation/deactivation cycle by GEF (guanine exchange factors) and GAP (GTPase activating proteins).

 The *RAS* gene family includes *H-RAS, K-RAS* and *N-RAS* and encodes for membrane-bound 21-kd guanosine-triphosphate-(GTP-) binding proteins regulating cell growth, differentiation and apoptosis by interacting with multiple effectors including mitogen-activated protein kinase (*MAPK*), phosphoinositide 3-kinase (*PI3K*) and signal transducer and activator of transcription (*STAT*) cascades (Figure 1). *RAS* proteins acquire transforming potential when a point mutation in the gene replaces an amino acid at position 12, 13 or 61 [19]. These mutations lead to forms of *RAS* with impaired GTPase activity, causing a constitutive activation of *RAS* signalling pathway. Mutations in *K-RAS* gene occur frequently in NSCLC [20], more frequently (20–30%) in adenocarcinoma and less frequently (about 7%) in squamous-cell carcinoma [21]. In NSCLC the vast majority of *K-RAS* mutations involve codons 12 or 13 and are usually associated with a history of tobacco use [22]. *K-RAS* mutations frequency varies among different ethnic groups, with lower frequency observed among Asians and higher frequency among African Americans compared to white Caucasians [23]. Recently *K-RAS* mutations have been detected in a significant proportion of never smoker NSCLC patients, with an incidence up to 15% [23]. Thus, unlike *EGFR *mutations, which occur more frequently in never smokers, presence of a *K-RAS* mutation cannot be predicted on the basis of smoking history alone.

## 3. *K-RAS* Mutation as Prognostic Biomarker

The role of* K-RAS* mutations as a prognostic factor in NSCLC remains controversial. Although some studies suggested a potential negative prognostic effect, other studies did not confirm any negative impact on survival for individuals harbouring a *K-RAS* mutation. More than 50 studies have been published, using different methods for *K-RAS* testing and with conflicting results (Table 1). In an ancillary study of JBR.10 trial, a phase III trial of adjuvant chemotherapy versus observation in resected NSCLC, among the 450 analyzed cases, 26% harboured a *K-RAS* mutation [24]. In the group of patients not treated with chemotherapy, *K-RAS* mutations were not prognostic for survival (*P* = 0.4). In the E4592 trial, another phase III trial of adjuvant chemotherapy versus observation in resected NSCLC, 24% of 184 assessable tumors were positive for *K-RAS* mutations [25]. The median survival of mutated and wild-type patients was not statistically different (30 and 42 months, resp. *P* = 0.38). Graziano et al. investigated the prognostic effect of *K-RAS* mutations in stage I and II resected NSCLC [26]. In the whole population, no statistical difference was found in OS for *K-RAS*-mutations-positive and negative patients (*P* = 0.33). Keohavong et al. found no association of *K-RAS* mutation and survival in 173 adenocarcinoma and adenosquamous NSCLC patients [27]. In another study, Lu et al. evaluated the prognostic role of a panel of six biomarkers including *K-RAS* mutations, in completely resected stage I NSCLC [28]. Patients were followed up for a minimum of 5 years; *K-RAS* mutations were detected in 34% of samples and were not associated with overall survival (*P* = 0.517). Conversely, Slebos reported a series of 69 surgically treated adenocarcinomas of the lung in which *K-RAS* codon-12 point mutations resulted in a negative prognostic factor for disease-free survival (*P* = 0.038) and overall survival (*P* = 0.002) [29]. This difference was consistent also after adjustment for factors such as stage, tumor size and differentiation. In a prospective series of 365 patients with resected early stage NSCLC treated at Massachusetts General Hospital, *K-RAS* mutations were found only in smokers and were associated with worse survival (*P* = 0.009, log-rank test) only in stage I disease, but not in the whole population [30]. In a Japanese study, Fukuyama et al. examined 159 cases of NSCLC for mutation at codon 12 of *K-RAS* gene and found 6.9% of mutated patients [31]. The *K-RAS* mutation positive group had a worse survival than the *K-RAS* negative group (*P* < 0.05). In another Japanese study, *K-RAS* mutations were detected in 8.3% of 144 patients [32]. The OS rate of NSCLC patients with wild-type *K-RAS* was better than that of patients whose tumors harboured mutations of *K-RAS* (*P* = 0.033). Miyake et al. analysed tumor tissue from 187 NSCLC patients, among which 8% harboured a *K-RAS* mutation [33]. In this study, patients with wild-type *K-RAS* had a significantly better survival rate than those with mutant *K-RAS* (*P* = 0.0369). In another study Marks et al. evaluated the prognostic role of *EGFR* and *K-RAS* in 296 resected lung adenocarcinomas [34]. Patients were stratified on *EGFR* and *K-RAS* mutation, *K-RAS* mutation or absence of *EGFR* and *K-RAS* mutation. In the absence of targeted therapies, 3-years OS was 90%, 76%, and 66% for patients with *EGFR* mutations, *EGFR/K-RAS* wild type and *K-RAS*-mutations, respectively. The difference in survival between *EGFR*-mutated group and *K-RAS* mutated group was statistically significant (*P* = 0.009). In 2005 a systematic review and meta-analysis of 28 studies including a total of 3620 patients showed that presence of *K-RAS* mutations confers a significantly worse prognosis, with a combined HR of 1.35 for OS in the random effect model [35]. In a subgroup analysis according to histology, *K-RAS* mutation resulted in a statistically significant prognostic factor for survival only for adenocarcinoma (HR 1.59).

Available data suggest that *K-RAS* mutations represent a negative prognostic factor particularly in patients populations with high incidence of *EGFR* mutations, such as in adenocarcinoma and in Asiatic patients. A possible explanation is that in adenocarcinoma and in Asiatic patients there is a high incidence of *EGFR* mutations that are considered a positive prognostic factor. In fact, in the study conducted by Marks et al., OS was significantly worse in lung adenocarcinomas with *K-RAS* mutations when compared to patients harbouring *EGFR* mutations [34].

## 4. *K-RAS* Mutation as Predictive Biomarker

### 4.1. Chemotherapy

Recent data suggested that *K-RAS* mutations may affect the outcome of NSCLC patients receiving chemotherapy (Table 2). In the adjuvant setting, data from the JBR10 trial suggested no benefit from adjuvant chemotherapy in *K-RAS* mutated patients (HR 0.95, *P* = 0.87) [24, 36]. In the LACE-BIO pooled analysis the prognostic and predictive role of *K-RAS* mutations was investigated in 1751 patients treated with adjuvant chemotherapy [37]. Among evaluable patients, 304 (19.7%) harboured *K-RAS* mutations with no effect on survival (HR 1.18, *P* = 0.09).

Several studies investigated the influence of *K-RAS* mutations on sensitivity to chemotherapy in advanced NSCLC. Camps et al. analyzed *K-RAS* status in plasma samples from 308 advanced NSCLC patients treated with cisplatin and docetaxel. No difference in PFS (5.4 versus 5.7 months, *P* = 0.2) or OS (10.0 versus 9.0 months, *P* = 0.5) was detected between *K-RAS* wild-type and *K-RAS* mutant patients [38]. Another study retrospectively analyzed 162 chemotherapy-naïve patients with locally advanced/metastatic NSCLC who received first-line chemotherapy [39]. Presence of *K-RAS* mutations did not affect response to chemotherapy (RR, 26.5% for *K-RAS* wild type versus 25% for *K-RAS* mutant; *P* = 0.87) nor time to progression (TTP, 4.2 months for *K-RAS* mutant versus 4.7 months for *K-RAS* wild type; *P* = 0.42). Furthermore, no significant difference in survival was detected between *K-RAS* wild type and *K-RAS*-mutated patients (14.5 versus 18.5 months for mutations positive and wild-type *K-RAS* patients, respectively; *P* = 0.52).

Overall, these data indicate that *K-RAS* mutations have no role in response prediction to standard chemotherapy in NSCLC and, therefore, such test should not be used in clinical practice.

### 4.2. EGFR-TKIs

**Figure 2:**
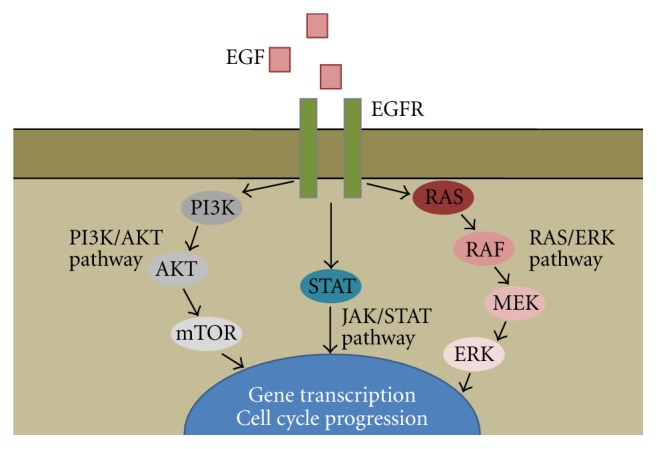
EGFR signalling pathway.

*K-RAS* is a critical downstream effector of the *EGFR* pathway (Figure 2). Therefore, there is a biologic rationale supporting the hypothesis that NSCLC tumors with *K-RAS* mutations are intrinsically resistant to *EGFR*-directed therapies. In fact, mutations in this gene may produce constitutive activation of the kinase that may overrule the inhibition of *EGFR* signaling. Initial studies in small cohorts of NSCLC showed lack of response to EGFR-TKIs in patients harboring *K-RAS* mutations [40–43]. Giaccone et al. analyzed *K-RAS* status in patients treated with frontline erlotinib and found that none of 10 mutated patients responded to anti-EGFR treatment [40]. Absence of response to erlotinib was reported in another phase II trial in elderly patients. In this study tissue samples from 41 patients were analyzed for *K-RAS* mutations and all the 6 mutated patients identified were refractory to erlotinib [41]. Pao et al. investigated the role of *K-RAS* mutations in 60 lung adenocarcinomas treated with gefitinib or erlotinib; *K-RAS* mutations were identified in 9 (24%) of 38 patients refractory to either drug, whereas no mutation was detected in 21 sensitive patients [42]. A retrospective analysis of *K-RAS* mutations in patients treated with EGFR-TKIs was conducted by Massarelli et al. In this study 16 (22.8%) of 70 patients had a *K-RAS* mutation and all of them (100%) had progressive disease during the treatment [43]. These studies suggested an association between *K-RAS* mutations and an absence of response to EGFR-TKIs. More recently, two meta-analyses showed that the presence of *K-RAS* mutations was associated with lack of response to EGFR-TKIs in NSCLC patients [44, 45]. Nevertheless, both meta-analyses were insufficient to determine the association between *K-RAS* status and PFS and OS.

Table 3 reports data on *K-RAS* mutational status and its relationship with survival in phase III trials with anti-EGFR therapy. In the TRIBUTE study, comparing chemotherapy and chemotherapy plus erlotinib, patients with *K-RAS* mutations had significantly shorter survival when treated with chemotherapy plus erlotinib, suggesting a possible detrimental effect of TKIs in patients harbouring such mutations [46]. The BR.21 trial, evaluating erlotinib versus placebo in second- and third-line setting, showed a survival advantage for erlotinib in the overall population (6.7 versus 4.7 months, HR 0.70; *P* < 0.001) [13]. Two hundred and six samples were available for *K-RAS* analysis and in 16% of cases a *K-RAS* mutation was detected. In the Cox model, the interaction between *K-RAS* mutation status and treatment suggested a lack of benefit from erlotinib in patients with mutations (*P* < 0.09). Importantly, on multivariate analysis, the presence of *K-RAS* mutation was not predictive for a differential treatment effect (*P* = 0.13) [47].

A potential benefit in survival produced by erlotinib in *K-RAS* mutated NSCLC has been reported in the SATURN trial, a large phase III trial randomizing 889 patients who did not progress after first-line chemotherapy, to receive erlotinib or placebo as maintenance treatment [14, 48]. Four hundred ninety-three (55.4%) tumor samples were analyzed for *K-RAS* mutations. Patients treated with erlotinib experienced longer PFS irrespectively of *K-RAS* mutational status, with a marginal even if not significant survival improvement in the *K-RAS* mutant population (HR 0.79). Another maintenance study, the ATLAS trial, evaluated maintenance treatment with bevacizumab plus placebo or erlotinib in metastatic NSCLC patients not progressing after 4 cycles of platinum-based chemotherapy. The addition of erlotinib significantly reduced the risk of progression (HR 0.72), with the highest benefit observed in the *EGFR* mutated patients [15]. Analysis of *K-RAS* mutations highlighted longer PFS for *K-RAS* wild type patients treated with bevacizumab/erlotinib (HR 0.66, log-rank *P* = 0.0105) but no difference for *K-RAS-*mutant patients (HR 0.92, log-rank *P* = 0.76) between the two arms. Finally, in the INTEREST study, a large phase III trial comparing gefitinib and docetaxel as second-line therapy in metastatic NSCLC, 18% of patients harboured *K-RAS* mutations [16, 49]. No differences in PFS and response rates were detected in both treatment arms according to *K-RAS* status, with no evidence of any differential survival effect (*P* = 0.51).

Therefore, although patients harbouring a *K-RAS* mutation do not respond to EGFR-TKIs, a minimal survival effect cannot be excluded. For such reason, at present, *K-RAS* testing is not recommended for precluding an EGFR-TKI therapy to any NSCLC patient.

### 4.3. Anti-EGFR Monoclonal Antibody

A second strategy aimed at inhibiting *EGFR* signalling is the use of monoclonal antibodies binding the extracellular domain of the receptor. Two large phase III trials investigated the combination of cetuximab, a human-murine chimeric anti-EGFR IgG monoclonal antibody, with chemotherapy versus chemotherapy alone [50, 51]. In the FLEX trial, 1125 patients with *EGFR* expressing advanced NSCLC were randomized to receive first-line cisplatin/vinorelbine with or without cetuximab [50]. The addition of cetuximab to chemotherapy led to a significant but clinically marginal survival improvement (11.3 versus 10 months, HR 0.87, *P* = 0.044) with an increased risk of toxicity, in particular febrile neutropenia. Similar results were observed in the BMS099 trial, a phase III trial that randomly assigned 676 chemonaïve NSCLC patients to carboplatin plus a taxane versus the same chemotherapy regimen plus cetuximab [51]. Notably, patients were enrolled into the study regardless of *EGFR* expression. Although a nonsignificant trend toward longer survival (9.6 versus 8.3 months HR 0.89, *P* = 0.17) was reported, the primary end point of improved PFS in the cetuximab arm was not met (4.4 versus 4.2 months, *P* = 0.2). Based on these studies results, European Medicine Agency (EMA) recently rejected cetuximab approval for advanced NSCLC. This decision clearly highlights the need for biomarkers useful in selecting patients potentially candidate to cetuximab therapy. A recent biomarker analysis of FLEX trial has highlighted a survival benefit in NSCLC patients overexpressing *EGFR* even in the absence of a PFS benefit [52]. This led to a new submission to EMA in March 2011.

 The lack of benefit from anti-EGFR monoclonal antibodies in colorectal cancer patients with *K-RAS* mutations has been demonstrated [53–55]. The status of *K-RAS* gene has been investigated even in FLEX and BMS099 trials. In the BMS099 study *K-RAS* mutant patients treated with cetuximab plus chemotherapy had a trend toward improved PFS and OS than those treated only with chemotherapy [56]. Similarly, in the FLEX trial, *K-RAS* gene testing failed to identify patients not benefiting from cetuximab and showed similar survival between *K-RAS *mutant and wild type patients regardless of treatment [57].

These results demonstrate that, unlike colorectal cancer case, the negative predictive value of *K-RAS* mutations in NSCLC remains unclear. A possible explication of the different role of *K-RAS* mutation in lung and colorectal cancer has been recently proposed. Danenberg et al. analyzed *K-RAS* mutation status in 2693 colorectal and lung specimens [58]. Surprisingly, different types of *K-RAS* mutations were detected in lung and colorectal cancer, with a significant predominance of DNA *K-RAS* transversions in NSCLC, likely linked to tobacco exposure. The ratio of base transversions to transitions was 3.27 versus 0.77 (*p* < 001) in NSCLC and colorectal cancer, respectively. Tobacco-carcinogenesis-associated G>T transversions (codon 12 GGT>TGT plus GGT>GGT) represented 61% of *K-RAS* mutations in NSCLC and 39% in colorectal cancer (*P* < 0.001). It is possible that the distinct mutation pattern and biological function may contribute to differences in predictive value for cetuximab therapy between NSCLC and colorectal cancer.

## 5. Conclusion

*K-RAS* mutation testing is a validated biomarker in clinical practice to predict anti-EGFR treatment outcome in colorectal cancer. In a significant fraction of NSCLC, particularly adenocarcinoma and smokers, a *K-RAS* mutation is detectable, but its prognostic and predictive role remains unclear. Although this event is generally considered associated to a worse prognosis and resistance to several drugs, including EGFR-TKIs, available data are conflicting, not supporting the use of *K-RAS* testing in clinical practice for selection of NSCLC.

Unfortunately, although *K-RAS* mutations are one of the most commonly occurring oncogene aberrations in human cancer, no specific treatment is currently available. A new hope for *K-RAS* mutant patients is represented by novel drugs currently under investigation in phase II and III trials [59]. More recently, scientists uncovered a crack in the molecular armor of *RAS*, a binding pocket of functional significance that could provide the long-sought attack point for a therapeutic agent [60]. Twenty-five compounds with affinity for binding to *RAS* oncoproteins were identified by nuclear magnetic resonance spectroscopy. Although all these compounds demonstrated weak affinity for *RAS* protein and inability to completely knock out the oncoprotein, they represent the first generation of *RAS* inhibitors, opening a new notable way for research of other compounds able to prevent *RAS* activation. While waiting for new drugs, the continuous collaboration between basic scientists and clinical researchers is the most relevant way to give hope to our cancer patients.

## Figures and Tables

**Table 1:** Prognostic value of *K-RAS* mutations.

Author	Total number of patients	*K-RAS* mutations (%)	Survival (*P*-value)
Tsao et al. [24]	450	26.0	0.4
Schiller et al. [25]	184	24.0	0.38
Graziano et al. [26]	213	16.4	0.33
Keohavong et al. [27]	173	32	0.74
Lu et al. [28]	94	34	0.52
Slebos et al. [29]	69	27.5	0.002∗
Nelson et al. [30]	365	22.1	0.009∗
Fukuyama et al. [31]	159	6.9	<0.05∗
Huang et al. [32]	144	8.3	0.03∗
Miyake et al. [33]	187	8.0	0.037
Marks et al. [34]	296	17%	NR

NR: not reported.∗Statistically significant.

**Table 2:** Predictive value of *K-RAS* mutations on overall survival in patients treated with chemotherapy.

Author	Setting	Total number of patients	*K-RAS* mutations (%)	Survival (HR/*P* value)
Tsao et al. [24]	Adjuvant	450	26.0	0.95/0.87
Tsao et al. [37]	Adjuvant	1751	19.7	1.18/0.09
Camps et al. [38]	Advanced	308	8.8	NR/0.51
Kalikaki et al. [39]	Advanced	162	22.6	NR/0.52

HR: hazard ratio; NR: not reported.

**Table 3:** *KRAS* and sensitivity to anti-EGFR agents in phase III trial.

Trial	Anti-EGFR agent	Total number of patients (*n*)	Patients tested for *KRAS *(*n*)	KRAS mutant *n* (%)	Survival in KRAS mutant (HR)
TRIBUTE [46]	Gefitinib	1079	264	55 (21)	2.1∗
BR. 21 [47]	Erlotinib	731	206	30 (15)	1.67
SATURN [48]	Erlotinib	889	493	90 (18)	0.79
ATLAS [15]	Erlotinib	768	NR	NR	0.92
INTEREST [49]	Gefitinib	1466	275	49 (18)	0.91
FLEX [57]	Cetuximab	1125	379	72 (19)	1.0
BMS099 [56]	Cetuximab	676	202	35 (17)	0.95

HR: hazard ratio; NR: not reported. ∗Statistically significant.

## Abbreviations

### Abbreviations

*NSCLC:*: Non-small-cell lung cancer
*EGFR:*: Epidermal Growth Factor Receptor
*ALK:*: Anaplastic Lymphoma Kinase
*TKIs:*: Tyrosine kinase inhibitors
*RR:*: Response rate
*PFS:*: Progression-free survival
*OS:*: Overall survival
*GTP:*: Guanosine triphosphate
*MAPK:*: Mitogen-activated protein kinase
*PI3K:*: Phosphoinositide 3-kinase
*STAT:*: Signal transducer and activator of transcription
*TTP:*: Time to progression
*EMA:*: European Medicine Agency
